# Trans-arterial radioembolization in intermediate-advanced hepatocellular carcinoma: systematic review and meta-analyses

**DOI:** 10.18632/oncotarget.11644

**Published:** 2016-08-26

**Authors:** Carla Rognoni, Oriana Ciani, Silvia Sommariva, Antonio Facciorusso, Rosanna Tarricone, Sherrie Bhoori, Vincenzo Mazzaferro

**Affiliations:** ^1^ Centre for Research on Health and Social Care Management (CERGAS), Bocconi University, Milan, Italy; ^2^ Evidence Synthesis and Modelling for Health Improvement (ESMI), University of Exeter Medical School, South Cloisters, St Luke's Campus, Exeter, UK; ^3^ Department of Surgery, Liver Surgery, Transplantation and Gastroenterology, Istituto Nazionale Tumori Fondazione IRCCS, National Cancer Institute of Milan, and University of Milan, Milan, Italy; ^4^ Department of Policy Analysis and Public Management, Bocconi University, Milan, Italy

**Keywords:** hepatocellular carcinoma, intermediate stage, advanced stage, trans-arterial radioembolization, meta-analysis

## Abstract

Trans-arterial radioembolization (TARE) is a recognized, although not explicitly recommended, experimental therapy for unresectable hepatocellular carcinoma (HCC).

A systematic literature review was performed to identify published studies on the use of TARE in intermediate and advanced stages HCC exploring the efficacy and safety of this innovative treatment.

Twenty-one studies reporting data on overall survival (OS) and time to progression (TTP), were included in a meta-analysis. The pooled post-TARE OS was 63% (95% CI: 56-70%) and 27% (95% CI: 21-33%) at 1- and 3-years respectively in intermediate stage HCC, whereas OS was 37% (95% CI: 26-50%) and 13% (95% CI: 9-18%) at the same time intervals in patients with sufficient liver function (Child-Pugh A-B7) but with an advanced HCC because of the presence of portal vein thrombosis. When an intermediate and advanced case-mix was considered, OS was 58% (95% CI: 48-67%) and 17% (95% CI: 12-23%) at 1- and 3-years respectively. As for TTP, only four studies reported data: the observed progression probability was 56% (95% CI: 41-70%) and 73% (95% CI: 56-87%) at 1 and 2 years respectively. The safety analysis, focused on the risk of liver decompensation after TARE, revealed a great variability, from 0-1% to more than 36% events, influenced by the number of procedures, patient Child-Pugh stage and treatment duration.

Evidence supporting the use of radioembolization in HCC is mainly based on retrospective and prospective cohort studies. Based on this evidence, until the results of the ongoing randomized trials become available, radioembolization appears to be a viable treatment option for intermediate-advanced stage HCC.

## INTRODUCTION

Hepatocellular carcinoma represents the third most common cause of cancer death, causing nearly 746,000 deaths per year in the world [1, 2]. In almost all cases, one risk factor can be identified alone or in combination with others, with cirrhosis being the most frequent of them [3]. Prognosis of patients with HCC is poor, with a ratio of mortality to incidence of 0.95 [2]. Indeed HCC represents a major global health problem, also considering that its incidence increases progressively with aging of population [4].

The Child-Pugh score is used to assess the prognosis of chronic liver disease, mainly cirrhosis [5]. Patients are classified according to different expected survivals from A to C (two year survival: A 85%, B 57%, C 35%) based on five clinical measures of liver disease (total bilirubin, serum albumin, prothrombin time, ascites, hepatic encephalopathy). The American and European Societies for the Study of the Liver endorse the Barcelona Clinic Liver Cancer (BCLC) classification system for staging and allocation to treatment in patients with HCC [6]. Using this classification, as others applied in the Asia-Pacific Countries, the therapeutic algorithm varies depending on the stage of disease, ranging from curative treatments (such as resection, ablation or transplantation) to palliation and best supportive care. Overall, only a minority of patients with HCC receive curative treatments and no more than 5% are eligible for liver transplantation [7].

In current practice, advanced stage patients present as such in 60-70% of cases while the rest progresses to this stage despite treatment of more precocious cancer presentations [6]. Prospective randomized controlled trials (RCTs) have shown that sorafenib prolongs survival in all subtypes of advanced HCC patients [8, 9] but side effects may lead to discontinuation of this treatment in up to 45% of patients. As a consequence, a significant number of advanced HCC patients may be precluded from the therapeutic benefit of sorafenib for related toxicities [10, 11].

In the intermediate HCC stage, a wide range of interventional loco-regional treatments are available and trans-arterial chemoembolization (TACE) variously administered, is considered as the standard of care [4, 6]. Also in advanced HCC - particularly when tumour extension is confined to the liver and when it is associated with a preserved hepatic function - the search for a tolerated loco-regional intervention able to challenge systemic therapy has been repeatedly investigated.

Trans-arterial radioembolization (TARE), often referred as a form of selective internal radiation therapy (SIRT) is a recognized, although not explicitly recommended therapy in several guidelines on clinical management of non-resectable HCC [4, 6, 12]. According to the latest release of the ESMO guidelines, however, TARE may compete with sorafenib in intermediate stage patients with prior TACE failure or advanced patients with tumoural macrovascular invasion (i.e. portal vein thrombosis, PVT) with no extra-hepatic spread and good liver function [12].

On technical grounds, TARE is a catheter-based interventional procedure that allows the emission of β-radiations at therapeutic levels directly into the tumour through its feeding arteries. Such a delivery mechanism, as for TACE, is aimed at minimizing the damage to the healthy liver parenchyma adjacent to the tumour. The main mechanism of action of TARE is a local brachytherapy and, unlike TACE, it doesn't result in microvascular embolization and tumour ischemia [13]. Devices for radioembolization are commercially available in both form of implantable glass (Therasphere^®^) or biocompatible resin-based (SIR-Spheres^®^) radioactive (Yttrium^90^ - Y^90^) spheres [13].

In the light of the increasing use of this innovative therapy in clinical practice, this study aims to systematically review the available evidence for the clinical efficacy and safety of TARE (glass or resin-based) in patients with intermediate-advanced stages HCC.

## RESULTS

**Figure 1:**
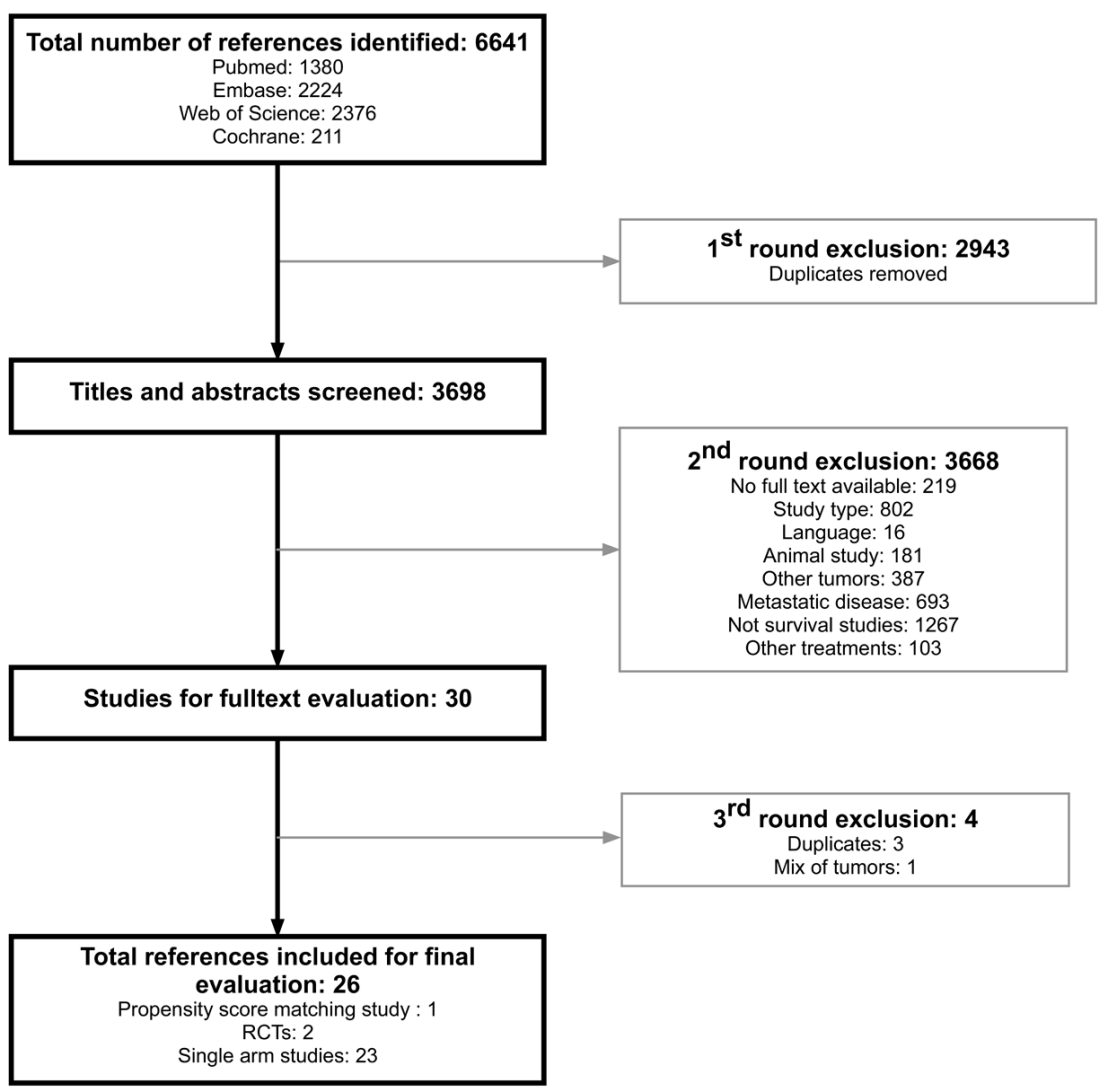
Study flow chart

Out of 6641 references screened, 26 studies met the selection criteria. The study selection process is summarized in Figure 1.

The vast majority of included studies were observational cohorts, however three comparative studies were found: two RCTs comparing TARE with either TARE + sorafenib [14] or TACE [15] and a propensity-score matching design study with sorafenib [16]. In Supplementary Table 1 a detailed description of all 26 studies is given.

A summary of the critical appraisal of TARE studies is reported in Supplementary Table 2.

All studies showed low to medium methodological quality. One study [20] did not present clearly inclusion and exclusion criteria and only 3 studies [15, 27, 36] were conducted in more than one centre. No therapy was reported as concurrently administered but in 7 studies follow-on treatments were described. Nine studies had a prospective design while dropouts were adequately described in only 3 studies.

### Outcome measures

Studies presenting OS or TTP data, stratified by presence or absence of PVT were all included in the meta-analyses except for two studies, one which did not report survival curves [17] and the second [37] which reported only survival data stratified by treatment dose, good or poor candidate groups and surgery vs. no surgery groups.

Studies considering patients with extrahepatic spread [26] or patients with deteriorated liver function (Child-Pugh C stage) [18] or patients awaiting liver transplantation [14] were excluded from the analysis.

Details on the 21 studies included in the quantitative analyses are listed in Table 1 together with patients and treatment characteristics.

**Table 1:** Characteristics of studies on radioembolization (TARE) included in the meta-analysis

Study	Study type	Patients characteristics	Child-Pugh (A-B-C)	% Patients with PVT	N arm TARE
Woodall 2009 [19]	Cohort	20 patients without PVT who received SIR, 15 patients with PVT who were treated. Of the PVT patients treated, 67% had portal VT, 7% had cava PVT, and 26% had both	Patients without PVT: 25% - 65% - 10% Patients with PVT: 20% - 53% - 27%	43%	35
Carr 2010 [20]	Cohort	HCC (with or without PVT) considered unsuitable for resection, RFA, or liver transplantation	NA	28%	99
Hilgard 2010 [21]	Cohort	Advanced HCC and liver cirrhosis (with or without PVT)	78% - 22% - 0%	31%	108
Kooby 2010 [22]	Cohort	Unresectable HCC (with or without PVT)	48% - 52% - 0%	52%	27
Salem 2010 [23]	Cohort	HCC (with or without PVT)	45% - 52% - 3%	43%	291
Tsai 2010 [24]	Cohort	HCC with main (n=10) or first (n=12) branch PVT	55% - 27% - 5% (NA 13%)	100%	22
Lambert 2011 [25]	Cohort	Patients suffering from HCC (with or without PVT) with the disease confined to the liver but not amenable to surgery, RFA or transplantation.	92% - 8% - 0%	7%	29
Sangro 2011 [27]	Cohort (retrospective)	Patients with unresectable HCC (with or without PVT)	83% - 17% - 0%	23%	325
Mazzaferro 2013 [28]	Phase II	Consecutive cohort of 52 patients with liver cirrhosis and HCC (with or without PVT) confined to the liver and not eligible to conventional curative treatments	83% - 17% - 0%	67%	52
Moreno-Luna 2013 [29]	Cohort (retrospective)	patients with unresectable HCC without PVT	87% - 13% - 0%	0%	61
Weng 2013 [30]	Cohort	HCC patients with PVT	NA	100%	149
Gramenzi 2014 [16]	Cohort (retrospective)	HCC patients (with or without PVT)	91% - 9% - 0%	41%	32
Khor 2014 [31]	Cohort	Patients with HCC (with or without PVT) unsuitable for surgical resection	59% - 38% - 3%	31%	103
Kwok 2014 [32]	Cohort	Inoperable HCC (with or without PVT)	83% - 17% - 0%	63%	30
Padia 2014 [33]	Cohort	Unresectable HCC (with or without PVT)	55% - 40% - 5%	60%	20
Saxena 2014 [34]	Cohort (retrospective)	HCC patients not amenable to curative surgical resection	67% - 22% - 2% (NA 9%)	NA	45
She 2014 [35]	Cohort	Patients with unresectable advanced HCC (with or without PVT)	94% - 6% - 0%	50%	16
El Fouly 2015 [36]	Cohort	HCCs classified as intermediate stage (BCLC B) without PVT	84% - 16% - 0%	0%	44
Kolligs 2015 [15]	RCT	Patients with unresectable HCC, Child-Pugh ≤B7, ECOG performance status ≤2 and ≤5 liver lesions without extrahepatic spread, without PVT	92% - 8% - 0%	0%	13
Ozkan 2015 [38]	Cohort	HCC patients (with or without PVT)	90% - 10% - 0%	41%	29
Soydal 2015 [39]	Cohort (retrospective)	Patients with unresectable HCC lesions	NA	NA	28

HCC = Hepatocellular carcinoma, PVT = portal vein thrombosis, TARE = trans-arterial embolization, BCLC = Barcelona Clinic Liver Cancer classification, ECOG = Eastern Cooperative Oncology Group, NA = not available

**Figure 2:**
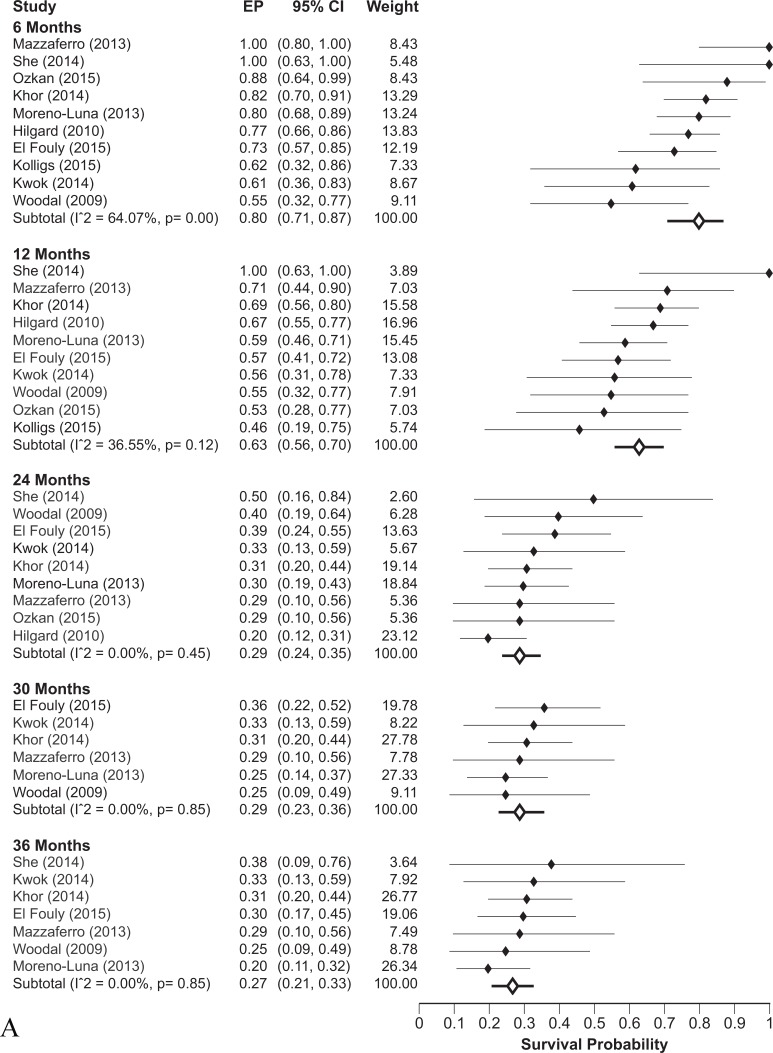

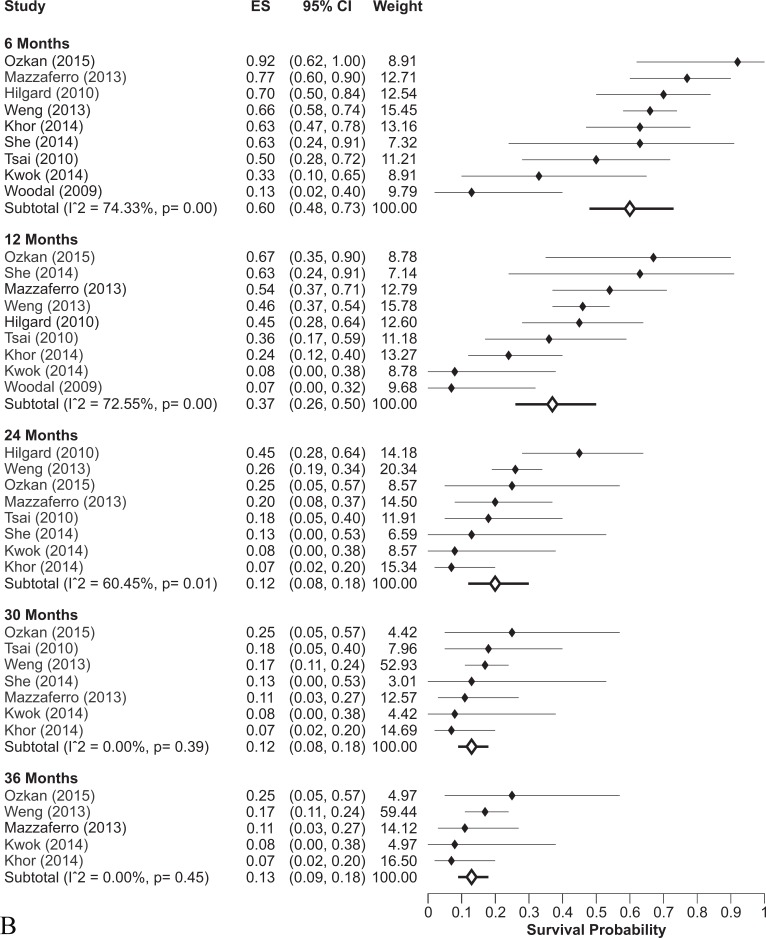
**A.** Overall survival rates at different follow-up times in intermediate-advanced HCC patients without PVT receiving TARE. **B.** Overall survival rates at different follow-up times in advanced HCC patients with PVT receiving TARE

The pooled OS was 63% (95% CI: 56-70%) and 27% (95% CI: 21-33%) at 1- and 3-years respectively, for the population receiving TARE at an intermediate-advanced stage (namely in unresectable HCC lacking demonstration of PVT: Figure 2A), whereas OS was 37% (95% CI: 26-50%) and 13% (95% CI: 9-18%) at the same follow-up times in patients with sufficient liver function but with an advanced HCC because of presence of PVT (Figure 2B).

When the entire population of patients receiving TARE was considered, with no stratification on PVT - i.e. with a case-mix of intermediate and advanced HCC - the OS was 58% (95% CI: 48-67%) and 17% (95% CI: 12-23%) at 1- and 3-years respectively (Supplementary Figure 1). In the cumulative analysis the percentage of patients with PVT was 38% while those with suboptimal liver function (Child-Pugh non-A) were 21%. Both these well-known prognostic variables likely influenced the observed results, although a direct evidence of that could not be inferred from the available data.

**Figure 3:**
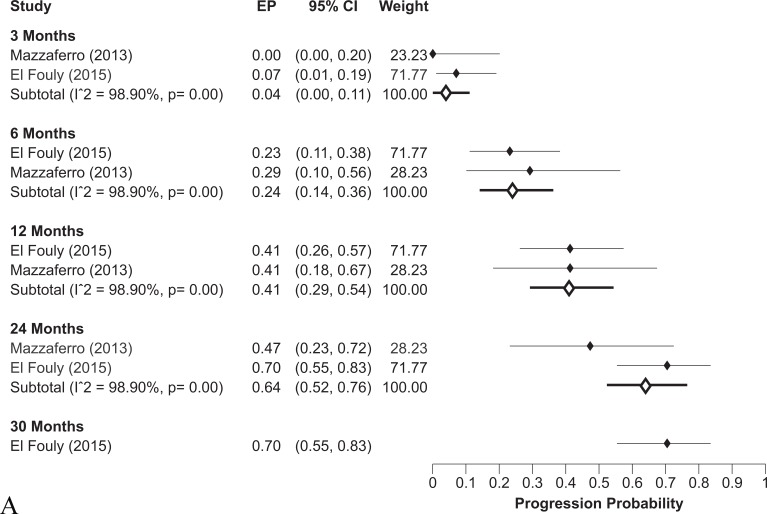

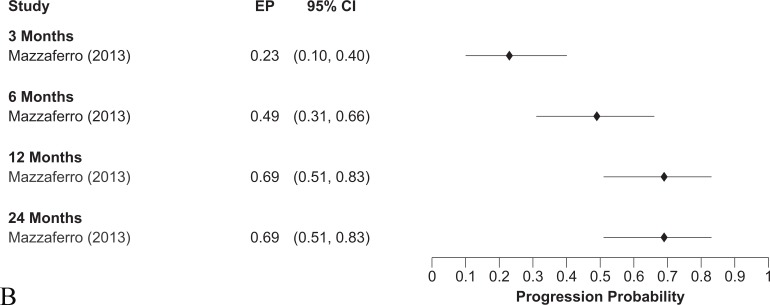
**A.** Probability of tumor progression at different follow-up times in intermediate-advanced HCC patients without PVT receiving TARE. **B.** Probability of tumor progression at different follow-up times in advanced HCC patients with PVT receiving TARE.

In terms of TTP, only four studies reported clear data: two of them had non-stratified TTP curves [21, 36], in one study data were stratified by Child-Pugh stage and BCLC classifications [23] and only one study stratified data on presence/absence of PVT [28]. Cumulative probability of progression in intermediate-advanced HCC lacking PVT at 1- and 2-years was 41% (95% CI: 29-54%) and 64% (95% CI: 52-76%) respectively (Figure 3A) while in advanced HCC carrying PVT TTP increased to 69% (95% CI: 51-83%) at both 1 and 2 years in a single study (Figure 3B). When the entire population of patients receiving TARE - with intermediate 55% and advanced 45% HCC - were pooled, the observed progression probability was 56% (95% CI: 41-70%) and 73% (95% CI: 56-87%) at 1 and 2 years respectively (Supplementary Figure 2).

All results were confirmed in fixed effect meta-analysis models (data not shown).

### Liver impairment after TARE

As described above, tumour progression after TARE was reported as a late event mainly due to deterioration of performance status and of Child-Pugh stage.

More specifically, the analysis on the adverse events related to TARE was focused on liver decompensation and/or failure following treatment. There was a large heterogeneity in definition of liver decompensation in the included studies and with that bias the percentage of patients suffering liver decompensation after TARE is reported in Table 2. In particular, TARE-related liver impairment events ranged from 0-1% in some studies to 36.5% in others depending on the number of treatments received, performance status, duration and type of pre-TARE treatments. Liver decompensation was more likely in patients with multiple pre-TARE therapies and borderline cirrhosis (HR: 3.2; 95% CI: 1.8-9.7) as previously reported [40]. Medium time from TARE to liver decompensation was 3 months (range: 1-6).

**Table 2:** Liver failure after TARE and study characteristics

Study	% of patients with liver impairment	Time from TARE (months)	Mean number of treatments per patient	Intervention	Mean dose	Previous treatments
Woodall 2009 [19]	10%	NC	PVT patients: 2; no PVT patients: 1.5	TARE (Therasphere)	median dose of 120 Gy (range 120-142)	NA
Carr 2010 [20]	NA	NA	Single, planned treatment, but 30% required a second treatment because of new, late appearing lesions	TARE (Therasphere)	from 135 to 150 Gy to the treated lobe	NA
Hilgard 2010 [21]	2.78%	1	1.47	TARE (Therasphere)	120±8 Gy	62% of patients were therapy-naive; the rest received prior local therapy with curative or palliative intent
Kooby 2010 [22]	22%	1	1.2±1.1	TARE (SIR-Spheres)	740-2220 MBq per lobe	NA
Salem 2010 [23]	19%	NC	1.8	TARE (Therasphere)	The median dose was 103 Gy per treatment (95% CI 99–108)	Resection 5%, RFA 2%, TACE 5%, Orthotopic liver transplantation 1%
Tsai 2010 [24]	13%*	1	1.45	Resin or glass microspheres	2.7 GBq (range 0.59-9.21)	NA
Lambert 2011 [25]	3%	in the weeks following treatment	1.2	TARE (Therasphere)	2.17 GBq, range 0.73 to 3.99 GBq	RFA 13.7%, Liver resection and radiolabelled Lipiodol 3.4%, Transplantation and subsequent sorafenib 3.4%
Sangro 2011 [27]	5.80%	3	1.08	TARE (Therasphere)	1.6 GBq (range 0.3-4.0)	TARE or TACE (27.4%), surgical resection or transplantation (18.2%), percutaneous ablation (9.2%)
Mazzaferro 2013 [28]	36.50%	6	1.12	TARE (Therasphere)	median 2.6 GBq (range 1.1-5.7); median dose to liver lobe 101 Gy per treatment (range 34-146)	RFA 13.5%, Liver resection 15.4%
Moreno-Luna 2013 [29]	0%		1.28	TARE (Therasphere)	The target dose of 80–150 Gy	NA
Weng 2013 [30]	NA		NA	TARE (Therasphere)	NA	NA
Gramenzi 2014 [16]	9% (grade 3-4) in the whole group of patients	6	Median tumour dose 263.2 Gy (range 16.6–1145.8); Total injected activity 1.83 GBq (0.45–2.41)	TARE (SIR-Spheres)	median tumor dose was 119.8 Gy (range, 31.4–420.2 Gy)	Resection 13.6 %, RFA 12.6%
Khor 2014 [31]	1%	NC	1.175	TARE (SIR-Spheres)	All patients received more than 2 GBq of Y90	NA
Kwok 2014 [32]	13%	3	NA	TARE (SIR-Spheres)	Median dose to treated segment 254Gy, median dose to the tumor 536 Gy	Ablation 20%, chemoembolization 5%, radioembolization 5%
Padia 2014 [33]	10%	NC	1	TARE (Therasphere)	1.49 GBq (range 0.34-2.50)	Liver resection 13%, transarterial chemoembolization or hepatic artery chemoinfusion 24%, ablative therapy 9%, at least one line of systemic chemotherapy 13%
Saxena 2014 [34]	4.40%	NC	NA	TARE (SIR-Spheres)	NA	NA
She 2014 [35]	6.30%	NC	NA	TARE (not specified)	NA	Resection 7%, RFA 11%, TACE 32%
El Fouly 2015 [36]	9.09%	1	1.4 ±0.6	TARE (Therasphere)	median 1.6±0.6 GBq	NA
Kolligs 2015 [15]	<5%	4	1	TARE (SIR-Spheres)	1.5 GBq (range 1-2.2)	34.50%
Ozkan 2015 [38]	0%		NA	resin or glass microspheres	1.5 ± 0.2 GBq	None of them had received prior treatment before SIRT
Soydal 2015 [39]	NA	NA	Treatment was applied to the right lobe in 22 patients and both lobes in 6 patients	TARE (resin)	NA	NA

* % on the number of treatments performed

TARE= trans-arterial radioembolization, TACE= trans-arterial chemoembolization, RFA= radio-frequency ablation, SIRT= selective internal radiation therapy, NC = not clear, NA = not available

## DISCUSSION

In the last decade trans-arterial radioembolization has emerged as a viable loco-regional treatment option for patients with unresectable hepatocellular carcinoma associated or not with portal vein thrombosis, although its use is still not formally recommended in clinical guidelines due to lack of prospective randomized studies. While large RCTs have been implemented and likely to be reported in the near future, the present study aimed at identifying the available evidence around the efficacy and safety of TARE through a systematic review of published studies conducted on patients with intermediate and advanced HCC.

We identified 23 observational cohort studies, two RCTs of TARE vs. TARE + sorafenib and TARE vs. TACE and one propensity score matching study comparing TARE vs. sorafenib. All studies were of intermediate to low methodological quality. After excluding papers not presenting the outcomes of interest (OS or TTP) in a suitable form for the meta-analysis, a pooled summary estimate on the 21 remaining studies confirmed the good efficacy performance of TARE in HCC unsuitable for curative treatment options.

Included studies suggest that median OS in patients receiving TARE for intermediate-advanced HCC falls in the range of 12-24 months, halved to 6-12 months should PVT be present. Pooled survival rates at 3-years after TARE were estimated as 13% (with PVT) and 27% (without PVT). These results are consistent with those presented in other published studies reporting median OS of 7-41.6 months in BCLC B to C patients [41].

Residual heterogeneity among studies - as measured by I^2^ - was observed particularly in TTP while being less evident for OS. This reflected different schedules and protocols for definition and identification of tumour progression, which is considered a secondary endpoint in the large majority of studies [42]. High heterogeneity was detected also when definition of liver decompensation related to TARE was taken into account; this forced us to compute and summarize data on adverse events only in a qualitative fashion. Despite such limitation, TARE-related liver toxicity was registered in less than 30% of patients with near to zero mortality directly related to the procedure and an average hospital stay claimed as shorter or equal compared to conventional chemoembolization [13].

This review has a number of limitations. First, only English language studies were included: considering that several clinical studies retrieved were conducted in non-English speaking countries, it is possible that some evidence on the clinical role of TARE has been overlooked. Moreover, individual level data used in the selected studies were not accessible. Individual rather than aggregate data, would have enriched the overall analysis [43].

As regards to the clinical outcomes, the number of papers in which post-progression survival after TARE is described is very limited, although sorafenib is used quite often as second-line therapy after TARE, thus affecting post-progression and overall survival. This was an unavoidable bias assumed to be equally distributed among the studies and therefore not considered as an exclusion driver. Inclusion of post-progression therapies reflected in fact the field practice adopted in the management of HCC patients.

The evidence that supports the use of radioembolization in HCC is based on retrospective or prospective cohort studies and no RCTs have been published comparing TARE with systemic therapies, in particular sorafenib, currently the mainstay for treating advanced HCC.

Large-scale randomized controlled trials with overall survival as a primary endpoint exploring this comparison have been started (SIRveNIB NCT01135056, SARAH NCT01482442, YES-P NCT01887717). These trials and future prospective randomized studies of TARE vs. suitable comparators, are crucial to provide direct evidence to evaluate comparative effectiveness because, at least in advanced HCC radioembolization may achieve overall survivals in the range of 6-10 months [27] competitive with the survival range (6.5-10.7 months) reported in the phase III clinical trials registering sorafenib as a standard of care.

Unfortunately, a pooled analysis focused on tumor response was not feasible due to high heterogeneity in reporting this outcome across the included studies. In fact, different response criteria (i.e. Response Evaluation Criteria In Solid Tumors - RECIST, mRECIST, World Health Organization - WHO criteria), imaging techniques, and time of radiological evaluation prevented a reliable assessment of this secondary endpoint. However, tumor response usually represents only a surrogate endpoint whereas OS, which is universally considered the primary outcome in oncological studies, was consistently assessed in our meta-analysis.

Among the studies comparing TACE *versus* TARE, two used Drug Eluting BeadsDEB-TACE) [26, 29] and five conventional TACE (cTACE) [15, 20, 22, 35, 36]. Although subgroup analysis according to the adopted TACE regimen was unfeasible due to the low number of studies, we know from previous cohort studies that DEB-TACE and cTACE lead to similar outcomes and comparable side effects in HCC patients [44, 45].

Observational data allow a preliminary assessment of the incremental costs vs. incremental effectiveness (i.e. cost-effectiveness) of TARE vs. sorafenib and help in this setting to assess the value of presumably similar treatment options in field practice. In a recent cost-effectiveness analysis of TARE vs. TACE, patients with advanced BCLC-C were found to benefit from radioembolization at an increased cost [46] while in patients with BCLC-A disease, who formally lack survival benefit from radioembolization, cost-efficacy could be obtained in some specific subgroups, such as PVT or technical unfeasibility of curative approaches. In the future, a cost-effectiveness analysis could be performed comparing TARE with the other available therapies, particularly sorafenib, to identify whether this procedure is cost-effective or not, and to profile HCC subgroups which could benefit from TARE at a reasonable cost. This analysis could be instrumental in helping policy-decision making, while additional post-marketing evidence is collected.

Given the findings of this systematic review, based on safety and efficacy data from included studies, until the results of the ongoing trials become available, radioembolization appears to be a viable treatment option for patients with intermediate-advanced stage HCC.

## MATERIALS AND METHODS

### Literature search

This review adopts the Preferred Reporting Items for Systematic Reviews and Meta-Analysis (PRISMA) statement [47]. In May 2015, a systematic search was conducted on PubMed, Embase, the Cochrane Library and Web of Science databases to retrieve clinical evidence on TARE for HCC. The search strategy was developed using the PICOS (Patient, Intervention, Comparator, Outcome, Study) framework. Boolean operators “AND” and “OR” were used to combine terms, while “NOT” operator was not included following Cochrane indications. Studies were considered if published in English and related to an adult population (≥18 years old). Reference lists of the retrieved articles were screened to find additional studies not identified through the original search.

### Selection criteria

Inclusion criteria are shown in Table 3. Given common indications emerging for Y^90^, the review was conducted with a focus on patients with intermediate and advanced HCC stages according to BCLC staging system (stages B and C), the latter particularly when presenting with PVT.

**Table 3:** PICOS inclusion criteria

**Population**	Studies considering adult population (≥18 years) with intermediate and advanced HCC* according with the BCLC staging system *studies reporting data stratified for PVT presence were categorized separately
**Intervention**	TARE using Y^90^-embedded glass or resin microspheres
**Comparator**	Not specified
**Outcome measures**	Overall survival; recurrence/progression-free survival, time to progression, safety
**Study types**	Validation studies; controlled clinical trials; randomized controlled trials; observational studies (case-report, letters, comments, editorials and non-systematic review were excluded)
**Availability**	English; full text
**Time and place**	Date and place limits were not set

HCC = Hepatocellular carcinoma, BCLC = Barcelona Clinic Liver Cancer classification, TARE = trans-arterial radioembolization, PVT = portal vein thrombosis

### Data extraction

Abstracts and full-text selection was conducted independently by two expert reviewers (CR, SS). In case of debate on study eligibility a third senior reviewer (AF) was involved to reach consensus. Data were extracted using a customized template developed in Microsoft Excel based on the PICOS statement. Information recorded included study features, participants', intervention's and comparator's characteristics, safety and efficacy outcomes.

### Data analysis

Data referring to TARE were retrieved from all comparative and non-comparative studies identified. Relevant efficacy outcomes (i.e. overall survival - OS - and time to progression - TTP - rates) were summarized and graphically presented through forest plots. When necessary, survival rates were derived from digitalization of Kaplan-Meier survival curves using the software Plot Digitizer 2.6.6©. OS was calculated as the difference between the date of the first treatment and the date of death from any cause, or last observation date in case of censoring. TTP was calculated from the first TARE treatment to the first progression at any site. Main adverse events, such as liver impairment, were tabulated and discussed qualitatively.

Survival rates for OS and TTP from different studies were pooled through a random effect meta-analysis of proportions with exact binomial confidence intervals [48]. A test on the summary effect measure is given, as well as a test for heterogeneity, also quantified using the I^2^ metric [49]: the higher the values (from 0% to 100%) the larger the heterogeneity across studies. Fixed effect meta-analyses were also performed to check the robustness of obtained estimates to alternative models specification. Results are displayed in forest plots according to follow-up time and relevant patient subgroups. Analyses were performed using Stata^®^ software (StataCorp, version 14).

### Quality assessment

The methodological quality of included studies was assessed according to a modified version of the National Health Service Centre of Review and Dissemination criteria [50]. Following this guidance, no scoring system was adopted; quality assessments were used for descriptive purposes [51].

## SUPPLEMENTARY MATERIALS FIGURES


