# Supplementary material for: Trans-arterial radioembolization in intermediate-advanced hepatocellular carcinoma: systematic review and meta-analyses
Source: Oncotarget. 2016 Aug 26;7(44):72343–55. doi: 10.18632/oncotarget.11644 (PMC5342166; doi:10.18632/oncotarget.11644)
Supplement: Supplementary file 1 [file oncotarget-07-72343-s001.pdf]

Trans-arterial radioembolization in intermediate-advanced hepatocellular carcinoma: systematic review and meta-analyses

Supplementary Material

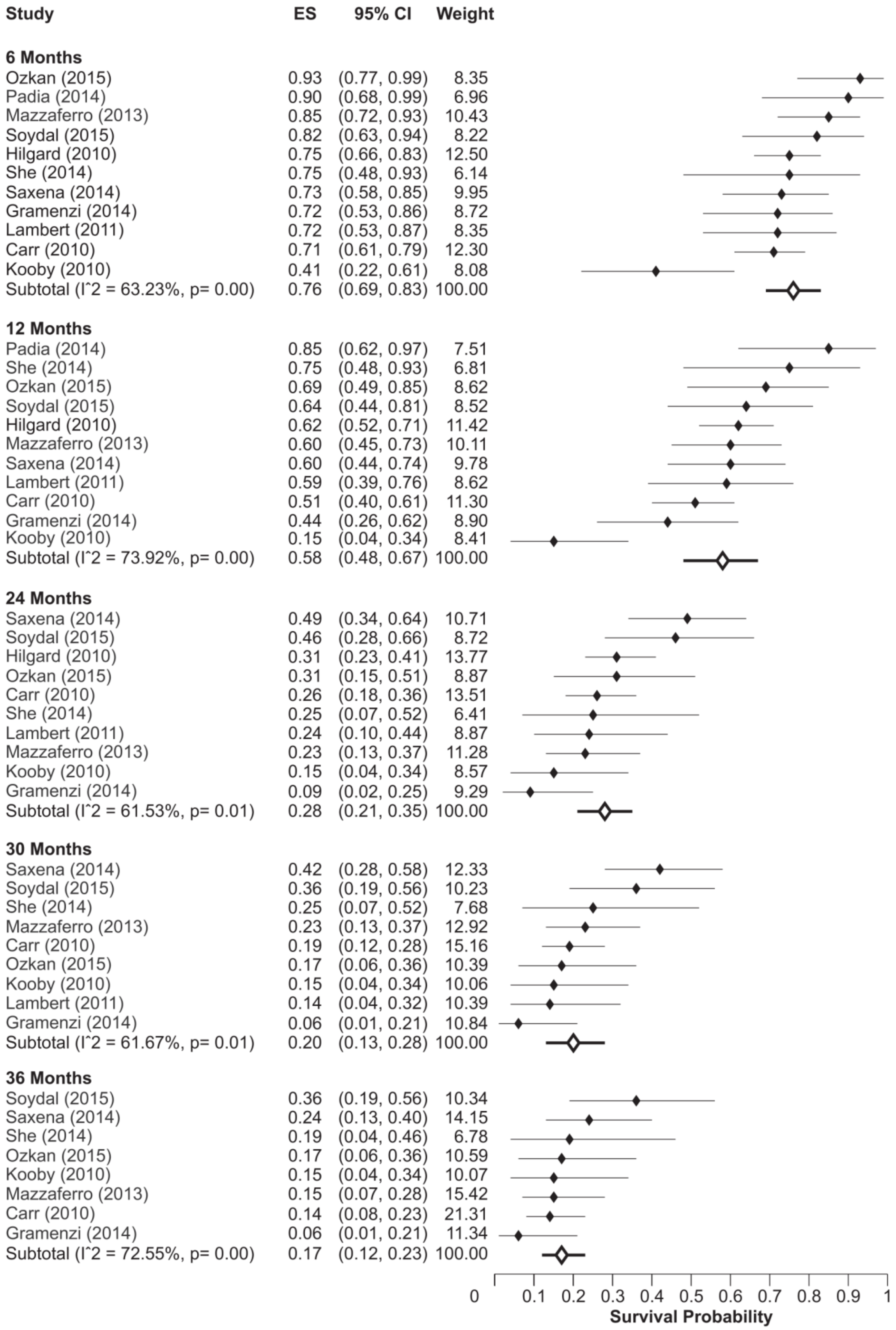

Supplementary Figure 1: Overall survival rates at different follow-up times in intermediate-advanced HCC patients receiving TARE

Study	EP	95% CI	Weight
**3 Months**			
El Fouly (2015)	0.07	(0.01, 0.19)	21.65
Hilgard (2010)	0.14	(0.08, 0.22)	52.80
Mazzaferro (2013)	0.15	(0.07, 0.28)	25.55
Subtotal (I^2 = 0.00%, p= 0.39)	0.12	(0.08, 0.18)	100.00
**6 Months**			
El Fouly (2015)	0.23	(0.11, 0.38)	29.04
Hilgard (2010)	0.39	(0.30, 0.49)	39.75
Mazzaferro (2013)	0.44	(0.30, 0.59)	31.21
Subtotal (I^2 = 62.61%, p= 0.07)	0.36	(0.25, 0.47)	99.99
**12 Months**			
El Fouly (2015)	0.41	(0.26, 0.57)	30.38
Mazzaferro (2013)	0.58	(0.43, 0.71)	31.97
Hilgard (2010)	0.66	(0.56, 0.75)	37.65
Subtotal (I^2 = 74.30%, p= 0.02)	0.56	(0.41, 0.70)	100.00
**24 Months**			
Mazzaferro (2013)	0.60	(0.45, 0.73)	32.48
El Fouly (2015)	0.70	(0.55, 0.83)	31.38
Hilgard (2010)	0.84	(0.76, 0.91)	36.14
Subtotal (I^2 = 83.01%, p= 0.00)	0.73	(0.56, 0.87)	100.00
**30 Months**			
El Fouly (2015)	0.70	(0.55, 0.83)	

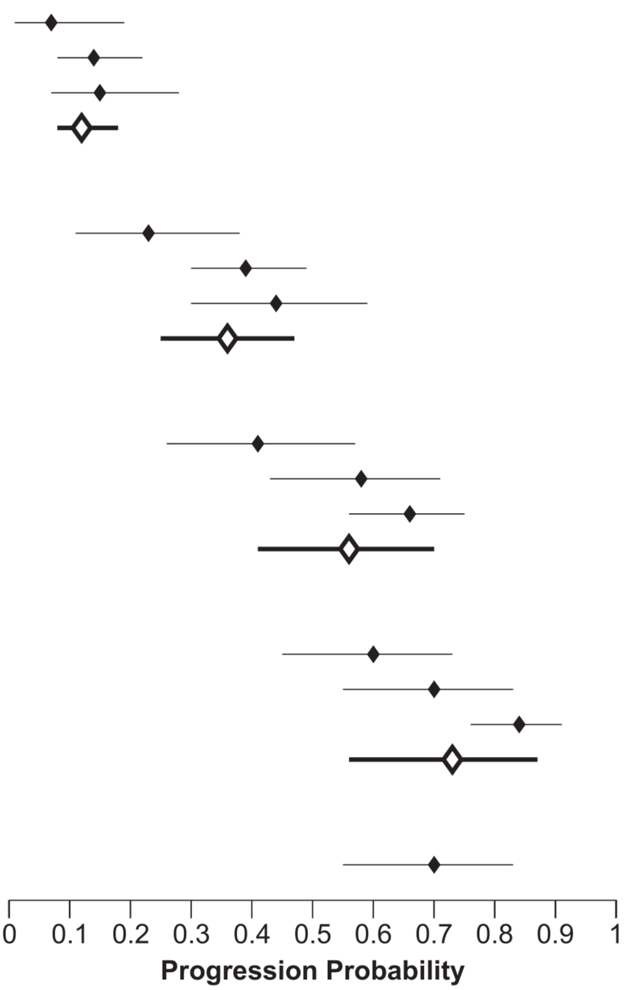

Supplementary Figure 2: Probability of tumor progression at different follow-up times in intermediate-advanced HCC patients receiving TARE
